# Schizophreniform disorder related hospitalizations – a Big Data analysis of a national hospitalization database

**DOI:** 10.1192/j.eurpsy.2022.821

**Published:** 2022-09-01

**Authors:** M. Pinho, A. Freitas, J.P. Ribeiro

**Affiliations:** 1 Centro Hospitalar do Tâmega e Sousa, Penafiel, Portugal, Department Of Psychiatry And Mental Health, Penafiel, Portugal; 2 CINTESIS - Center for Health Technology and Services, Faculty of Medicine, University of Porto, Department Of Community Medicine, Information And Health Decisions Sciences, Braga, Portugal; 3 CINTESIS - Center for Health Technology and Services, Faculty of Medicine, University of Porto, Department Of Community Medicine, Information And Health Decisions Sciences, Porto, Portugal

**Keywords:** Big Data, Administrative Database, hospitalization, Schizophreniform

## Abstract

**Introduction:**

Patients with schizophreniform disorder(SD) and schizophrenia present similar symptoms, however, SD has a shorter duration, varying between at least 1 month and 6 months.

**Objectives:**

To describe and analyse Schizophreniform disorder related hospitalizations in a national hospitalization database.

**Methods:**

We performed a retrospective observational study using a nationwide hospitalization database containing all hospitalizations registered in Portuguese public hospitals from 2008 to 2015. Hospitalizations with a primary diagnosis of schizophreniform diso72.1-der were selected based on International Classification of Diseases version 9, Clinical Modification (ICD-9-CM) code of diagnosis 295.4x. Birth date, sex, residence address, primary and secondary diagnoses, admission date, discharge date, length of stay (LoS), discharge status, and hospital charges were obtained. Comorbidities were analysed using the Charlson Index Score. Independent Sample T tests were performed to assess differences in continuous variables with a normal distribution and Mann-Whitney-U tests when no normal distribution was registered.

**Results:**

In Portuguese public hospitals, a total of 594 hospitalizations with a primary diagnosis of Schizophreniform disorder were registered during the 8-year study period. Most were associated to the male sex patients, 72.1% (n=428). The mean age at admission was 35.99 years and differed significantly between sexes (males - 34.44; females- 40.19; p<0.001). The median LoS was 17.00 days and the in-hospital mortality was 0.5% (n=3). Only 6.1% (n=36) of the hospitalization episodes had 1 or more registered comorbidities.

**Conclusions:**

Hospitalizations with a primary diagnosis of Schizophreniform disorder occur more frequently in young male patients. This is the first nationwide study analysing all hospitalization episodes in Portugal.

**Disclosure:**

No significant relationships.

